# Rotation Dynamics of Star Block Copolymers under Shear Flow

**DOI:** 10.3390/polym10080860

**Published:** 2018-08-03

**Authors:** Diego Jaramillo-Cano, Christos N. Likos, Manuel Camargo

**Affiliations:** 1 Faculty of Physics, University of Vienna, Boltzmanngasse 5, 1090 Vienna, Austria; diego.jaramillo@univie.ac.at (D.J.-C.); christos.likos@univie.ac.at (C.N.L.); 2 CICBA, Universidad Antonio Nariño—Campus Farallones, Km 18 vía Cali-Jamundí, Cali 760030, Colombia

**Keywords:** star block-copolymers, hybrid mesoscale simulation technique, rotational frequency, laboratory frame, Eckart frame, geometrical approach

## Abstract

Star block-copolymers (SBCs) are macromolecules formed by a number of diblock copolymers anchored to a common central core, being the internal monomers solvophilic and the end monomers solvophobic. Recent studies have demonstrated that SBCs constitute self-assembling building blocks with specific softness, functionalization, shape and flexibility. Depending on different physical and chemical parameters, the SBCs can behave as flexible patchy particles. In this paper, we study the rotational dynamics of isolated SBCs using a hybrid mesoscale simulation technique. We compare three different approaches to analyze the dynamics: the laboratory frame, the non-inertial Eckart’s frame and a geometrical approximation relating the conformation of the SBC to the velocity profile of the solvent. We find that the geometrical approach is adequate when dealing with very soft systems, while in the opposite extreme, the dynamics is best explained using the laboratory frame. On the other hand, the Eckart frame is found to be very general and to reproduced well both extreme cases. We also compare the rotational frequency and the kinetic energy with the definitions of the angular momentum and inertia tensor from recent publications.

## 1. Introduction

Polymer solutions have an important role from both the fundamental and applied point of views. The addition of a small amount of polymers to a liquid can be use to tune the stability and rheological properties on multiple commercial systems as paints, pharmaceutical products, food and oils. As a consequence of the polymer flexibility, a field flow can provoke large conformational changes, which in turn influence the flow field. In this way, understanding the coupling between the conformational and dynamical properties of isolated polymers immersed in a field flow is an important first step to elucidate the rheological behavior of (dilute and semi-dilute) polymer solutions [1,2]. To date, there has been a considerable amount of work on the response of flexible polymers with different architectures (e.g., linear, ring, hyperbranched and star polymers) to shear stress, which has revealed generic and specific properties of such systems. On top of experimental techniques, the development of simulation methods allowing one to efficiently couple the solvent particles and monomers, a wide spectrum of behaviors has been found regarding the average deformation and the orientation as a function of the shear rate, as well as multiple dynamic responses [3,4,5,6,7,8,9]. The latter encompass stretching and recoil, tumbling, tank-treading, rupture and collapse of polymers and ultimately determine the (complex) viscoelastic response of dilute bulk phases.

In this work, we consider the dynamics of isolated star block copolymers (SBCs), which can be exploited as versatile building blocks as they self-assemble into structures with one or multiple clusters of their solvophobic segments, i.e., they behave as self-associating patchy particles, featuring tunable softness, functionalization, shape and flexibility [10,11]. Recently, the structural properties of isolated SBCs under (linear) shear flow were analyzed by means of particle-based multiscale simulations for a wide set of parameters, which include the functionality of the star, the amphiphilicity degree, the solvent quality and the shear rate. In particular, the formation of attractive patches on the SBC corona as a function of the shear rate was analyzed. Three mechanisms of patch reorganization under shear were identified, which determine the dependence of the patch numbers and orientations on the shear rate, namely free arms joining existing patches, the fusion of medium-sized patches into bigger ones and the fission of large patches into two smaller ones at high shear rates [12].

Along with these studies, the dynamic behavior of single SBCs must be considered to gain some insights into the influence of these patch rearrangements on the rheology of dilute suspensions. Motivated by a very recent work on the rotational dynamics of star polymers in shear flow [13,14], this work focuses on the dynamics of sheared SBCs analyzed by means of the so-called Eckart frame, which allows one to separate pure rotational and vibrational motions. We show that SBCs display a richer structural and dynamical behavior than athermal star polymers in a shear flow, and therefore, they are also interesting candidates to tune the viscoelastic properties of complex fluids. The rest of the manuscript is organized as follows: In Section 2, we present the model and the employed tools. In Section 3, the simulation results are displayed, and the ensuing dynamic properties are discussed. Finally, in Section 4, we summarize and draw our conclusions.

## 2. Materials and Methods

### 2.1. Model and Simulation Method

#### 2.1.1. Coarse-Grained Model for the Star Block Copolymer

As mentioned above, the dynamics of a single SBC immersed in a sheared (Newtonian) solvent is studied by means of a hybrid multiparticle collision dynamics-molecular dynamics (MPCD-MD) method, as described in detail in [11,12]. Briefly, the star polymer and the solvent particles are modeled at a coarse-grained level. Each arm of the SBC is represented as a bead-spring chain having $N_{A}$ inner and $N_{B}$ outer monomers, thereby defining the degree of polymerization $N_{\mathrm{pol}}=N_{A}+N_{B}$ and the amphiphilicity $\alpha =N_{B}/N_{\mathrm{pol}}$. The monomers are represented as soft spheres of diameter σ and mass *M* interacting through pair potentials $V_{AA}\left(r\right)=V_{AB}\left(r\right)=V\left(r;0\right)$ and $V_{BB}\left(r\right)=V\left(r;\lambda \right)$, where:

(1) $$V\left(r;\lambda \right)=\left\{\begin{array}{cc} V_{0}\left(r\right)+\left(1-\lambda \right)\epsilon & r\leq r_{c}\\ \lambda V_{0}\left(r\right) & r>r_{c}. \end{array}\right.$$

Here, $V_{0}\left(r\right)=4\epsilon \left[{\left(\sigma /r\right)}^{48}-{\left(\sigma /r\right)}^{24}\right]$, $r_{c}=2^{1/24}\sigma$, *r* is the monomer-monomer distance, and λ is an attraction-coupling constant. The latter allows us to tune the solvent quality for the B-monomers; as explained in [11]. In particular, increasing the value of λ enhances the attraction between the B-monomers. Sufficiently large values of this parameter, λ>0.92, are equivalent to considering that a homopolymer made of B-monomers is below its θ-temperature. The bonding between connected monomers is introduced by an FENEpotential:

(2) $$U_{\mathrm{bond}}\left(r\right)=-\frac{1}{2}K\;R_{0}^{2}\;\ln\left[1-{\left(\frac{r}{R_{0}}\right)}^{2}\right],$$

where $K=30(\epsilon /\sigma^{2})$ and $R_{0}=1.5\sigma$.

#### 2.1.2. Multiparticle Collision Dynamics and Molecular Dynamics

**Figure 1:**
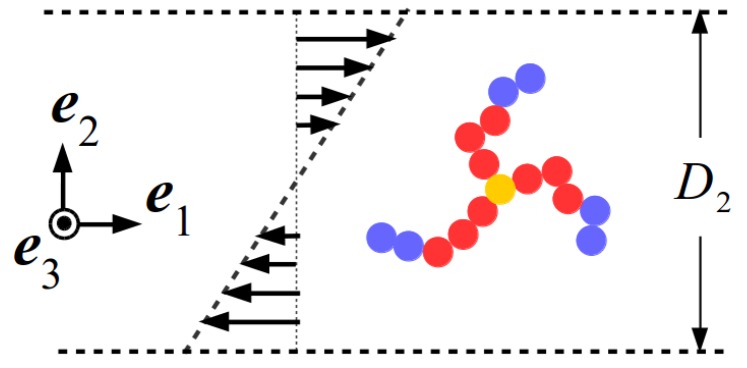
Schematic illustration of the simulation setup, demonstrating the shear (${\hat{e}}_{1}$), gradient (${\hat{e}}_{2}$) and vorticity (${\hat{e}}_{3}$) directions of planar, Couette flow. Yellow, red and blue spheres correspond respectively to the star core, solvophilic (A type) monomers and solvophobic (B type) monomers.

Multi-particle collision dynamics (MPCD) was employed to mesoscopically simulate the solvent [15,16]. The latter is assumed to be composed of $N_{s}$ point-like particles of mass *m*, whose dynamics follows two steps: a streaming step, in which the solvent particles move ballistically, and a collision step, in which the solvent particles exchange linear momentum. To do that, particles are sorted into cubic cells with length *a*, and their relative velocities with respect to the cell center-of-mass are rotated by an angle χ around a random axis [6,15,16]. The number of solvent particles per MPCD-collision cell is ρ=5, and their mass is m=M/ρ, serving as the unit of mass of the simulation; a convenient timescale is defined as $\tau =\sqrt{m\sigma^{2}/\epsilon }$. In what follows, we choose m=σ=ϵ=1, setting thereby the units of mass, length and energy, respectively; accordingly, τ serves as the unit of time. For the temperature *T*, we choose the value $k_{B}T=\epsilon /2$, where $k_{B}$ is the Boltzmann constant. The remaining MPCD-parameters were set as follows: the time between collisions is $\Delta t_{\mathrm{mpcd}}=0.1\tau$, the rotation angle is $\chi =130^{\circ }$ and the cell size a=σ, making the presence of two monomers in the collision cell very unlikely. Lees–Edwards boundary conditions were used to generate a shear velocity field $v\left(x_{2}\right)=\dot{\gamma }\;x_{2}\;{\hat{e}}_{1}$, characterized by the shear rate $\dot{\gamma }$, as schematically depicted in Figure 1.

In the MD-section of the hybrid technique, time evolution of the monomers follows the Newtonian equations of motion, which are integrated by means of the velocity-Verlet scheme [17] with an integration time step $\Delta t_{\mathrm{md}}=10^{-3}\tau$. The coupling between the monomers of the SBC and the solvent particles is achieved during the collision step, in which the former are included as point particles in the evaluation of the center-of-mass velocity of each cell, and their velocities are also randomly rotated. This interaction is strong enough to keep the monomers at the desired temperature, once a thermostat for the solvent particles has been introduced, which in the present case corresponds to a cell-level, Maxwell–Boltzmann scaling [18]. During the collision step, mass, momentum and energy are conserved, leading to correlations among the particles and giving rise to hydrodynamic interactions. As a dimensionless measure of the shear rate, we consider the Weissenberg number Wi, which is the product of the shear rate with the longest relaxation time of the polymer. For the latter, we take the longest Zimm relaxation time $\tau_{Z}$ of a polymer with $N_{\mathrm{pol}}$ monomers, which is given by the expression [6,19]:

(3) $$\tau_{Z}=\frac{\eta_{s}}{k_{B}T}\sigma^{3}N_{\mathrm{pol}}^{3\nu },$$

where $\eta_{s}$ is the (MPCD) solvent viscosity and ν=3/5 is the Flory exponent for self-avoiding chains. We obtain $\tau_{Z}≃1.3\times 10^{4}\;\tau$ for the specific choices of the MPCD collision parameters and the value $N_{\mathrm{pol}}=40$ employed here. Although we neglect any dependence of the relaxation time on star functionality *f* and attraction strength λ along the arms, the results justify a posteriori the choice of a common relaxation time, in the sense that we are able to obtain results for the shape parameters that mostly collapse on one another when plotted against $Wi=\dot{\gamma }\tau_{Z}$.

We performed a total of 14 independent runs with different initial conditions for each set of parameters {f,α,λ} investigated, covering a broad range of Wi, from the linear (Wi≲1) all the way to the strongly nonlinear ($Wi≃10^{3}$) regime. We focus on the following three particular sets of parameters: {f,α,λ}={12,0.3,1.0} (Case 1), {15,0.5,1.1} (Case 2) and {18,0.7,1.1} (Case 3). According to our previous study, these parameters represent the typical trends found in regard to the patchiness of the SBCs, namely: no patches are formed; several patches are formed having a small population; and few (one or two) bulky patches are formed [12]. For each run, a preparation cycle of $5\times 10^{6}$ MD steps was executed in the first place, which was long enough for the SBC to reach its stationary state, and then, a production cycle of $1.5\times 10^{7}$ MD steps took place. Depending on the shear rate, the simulation box has dimensions of $60\sigma \leq D_{1}\leq 110\sigma$ and $D_{2}=D_{3}=60\sigma$. Configuration data were saved every $N_{\mathrm{save}}=2\times 10^{4}$ MD steps during the production cycle. As in this work there exist various physical systems and they are looked at from various frames of reference and at different levels of approximations as regards their rotational dynamics, we use in what follows a number of abbreviations, whose meaning is summarized in Table 1 below.

### 2.2. Rotational Dynamics

Soft colloids and polymers under shear flow deform and undergo a succession of complex motion patterns, such as tumbling and tank-treading, which are hard to decouple from one another and analyze quantitatively. Recent studies aimed at a better understanding of the complex dynamics of (athermal) star polymers in shear flow have demonstrated that Eckart’s formalism allows one to separate correctly the different characteristic motions of the polymer, i.e., pure rotation, vibration with no-angular momentum and vibrational angular momentum [13,14]. In the following, a brief description of this formalism is given, which will be subsequently employed to analyze our simulation results.

#### 2.2.1. Laboratory Frame

Here, the frame of reference is fixed in space, and it is customarily and conveniently chosen in such a way that the first axis lies along the flow direction, the second along the gradient direction and the third along the vorticity direction, as shown in Figure 1. Taking $r_{k}$ and ${\dot{r}}_{k}$ as the position and the velocity of the *k*-th monomer in the laboratory frame of reference, the total angular momentum of a star polymer with respect to its center of mass is, by definition:

(4) $$L=M\sum_{k=1}^{N_{\mathrm{mon}}}\Delta r_{k}\times \Delta {\dot{r}}_{k},$$

with $k=1,\cdots ,N_{\mathrm{mon}}=f\;N_{\mathrm{pol}}+1$, $N_{\mathrm{mon}}$ the total number of monomers, $\Delta r_{k}=r_{k}-r_{\mathrm{cm}}$ and $\Delta {\dot{r}}_{k}={\dot{r}}_{k}-{\dot{r}}_{\mathrm{cm}}$. Here, $r_{\mathrm{cm}}$ and ${\dot{r}}_{\mathrm{cm}}$ are, respectively, the position and the velocity of the center of mass, i.e.,

(5) $$r_{\mathrm{cm}}=\frac{1}{N_{\mathrm{mon}}}\sum_{k=1}^{N_{\mathrm{mon}}}r_{k}.$$

The time evolution of the *k*-th monomer position can be evaluated as [13,14,20,21]:

(6) $${\dot{r}}_{k}={\dot{r}}_{\mathrm{cm}}+\left(\omega \times \Delta r_{k}\right)+{\tilde{v}}_{k},$$

where ${\tilde{v}}_{k}$ denotes a purely vibrational motion, which is angular momentum-free in the laboratory frame, i.e., ${\tilde{v}}_{k}$ and $\Delta r_{k}$ are parallel (cf. Equation (4)). The angular frequency ω can be expressed as:

(7) $$\omega =J^{-1}L,$$

with the components of the moment of inertia tensor J being defined as:

(8) $$J_{\mu \nu }=M\sum_{k=1}^{N_{\mathrm{mon}}}\left[\Delta r_{k}^{2}\delta_{\mu \nu }-\Delta r_{k,\mu }\Delta r_{k,\nu }\right]\;\left(\mu ,\nu =1,2,3\right),$$

with $\delta_{\mu \nu }$ the Kronecker delta and $r_{k,\mu }$ the μ-th component of the position vector of the *k*-th monomer. In the case of rigid-body motion, $\tilde{v}=0$ and ω coincides with the rotational angular velocity.

The full kinetic energy $E_{\mathrm{kin}}$ of the sheared polymer results from Equation (6) and reads as:

(9) $$\begin{array}{ccc} E_{\mathrm{kin}} & = & \frac{1}{2}M\sum_{k}{\dot{r}}_{k}\cdot {\dot{r}}_{k}\\ & = & \frac{1}{2}M_{s}\;{\dot{r}}_{\mathrm{cm}}\cdot {\dot{r}}_{\mathrm{cm}}+\frac{1}{2}\omega \cdot J\cdot \omega +\frac{1}{2}M\sum_{k}{\tilde{v}}_{k}\cdot {\tilde{v}}_{k}, \end{array}$$

where $M_{s}=N_{\mathrm{mon}}M$ is the total mass of the polymer. The three terms in the r.h.s of Equation (9) represent the translational, rotational and vibrational contributions to the kinetic energy, respectively. We emphasize, though, that the velocity contribution ${\tilde{v}}_{k}$ in the motion of a monomer is not the only vibrational contribution, but just the one that does not contribute to the (instantaneous) angular momentum; there are, in general, additional vibrational contributions included in ω. Therefore, ω is the apparent angular velocity, and it is not possible to separate rotation from vibrational with angular momentum motion within the lab frame.

#### 2.2.2. Eckart Frame

Eckart’s formalism makes use of a non-inertial frame, which co-rotates with the polymer at angular velocity Ω (see Equation (15) below) [22,23]. The first step to build up the Eckart frame is to choose one initial configuration of the SBC as a reference, accompanied by an initial frame of reference spanned by the basis vectors $\left\{f_{1}\left(0\right),f_{2}\left(0\right),f_{3}\left(0\right)\right\}$. The origin of this frame is located at the center of mass of the chosen reference configuration of the polymer, and as a matter of convenience, the three axes $\left\{f_{1}\left(0\right),f_{2}\left(0\right),f_{3}\left(0\right)\right\}$ also coincide with the orientation of the laboratory frame. Due to the choice of the origin, in this system of coordinates, the position vectors of the monomers at time t=0, $\left\{a_{k}=\Delta r_{k}\left(0\right);k=1,2,\ldots ,N_{\mathrm{mon}}\right\}$, satisfy the relation:

(10) $$\sum_{k=1}^{N_{\mathrm{mon}}}a_{k}=0.$$

This reference configuration is frozen and co-rotates with the Eckart frame of reference, the latter evolving with time as explained below. In the second step of the process, the unit base (column) vectors $\left\{f_{1}\left(t\right),f_{2}\left(t\right),f_{3}\left(t\right)\right\}$ of the instantaneous Eckart frame are evaluated. To achieve that, the vectors:

(11) $$F_{\mu }\left(t\right)=M\sum_{k}a_{k,\mu }\Delta r_{k}\left(t\right)\;\left(\mu =1,2,3\right),$$

are introduced, which are completely defined in terms of the instantaneous positions $\Delta r_{k}\left(t\right)$ and the Cartesian components $a_{k,\mu }$ of the reference position vectors $a_{k}$ for each monomer. In what follows, we drop the explicit time-dependence from the notation of the various vectors. The right-handed triad of unit vectors $\left\{f_{1},f_{2},f_{3}\right\}$ is determined as:

(12) $$\left[f_{1},f_{2},f_{3}\right]=\left[F_{1},F_{2},F_{3}\right]F^{-1/2},$$

where the elements of the symmetric (Gram) matrix F are defined as ${\left[F\right]}_{\mu \nu }=F_{\mu }\cdot F_{\nu }$ and $F^{-1/2}F^{-1/2}=F^{-1}$. In this way, the position vector $c_{k}$ of the *k*-th monomer in the co-rotating reference configuration, decomposed onto the unit vectors of the rotating Eckart frame of reference, is given by:

(13) $$c_{k}=\sum_{\mu =1}^{3}a_{k,\mu }f_{\mu },$$

the coefficients $a_{k,\mu }$ being fixed, time-independent quantities set by the reference configuration and the triad $\left\{f_{1},f_{2},f_{3}\right\}$ depending on time as explained above. In this way, the $c_{k}$ are constant vectors when looked at from within the rotating Eckart frame and describe the original, rigid configuration.

**Figure 2:**
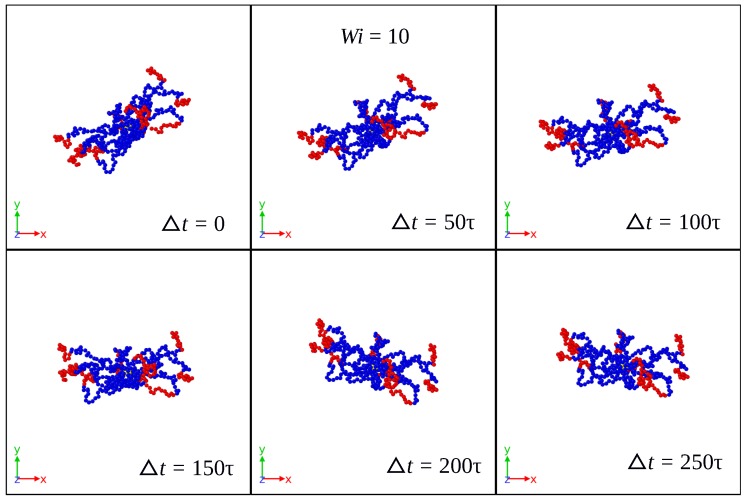
Time evolution of the (fixed) reference configuration in Eckart’s frame as seen in the laboratory frame for Case 1 and Wi=10.

**Figure 3:**
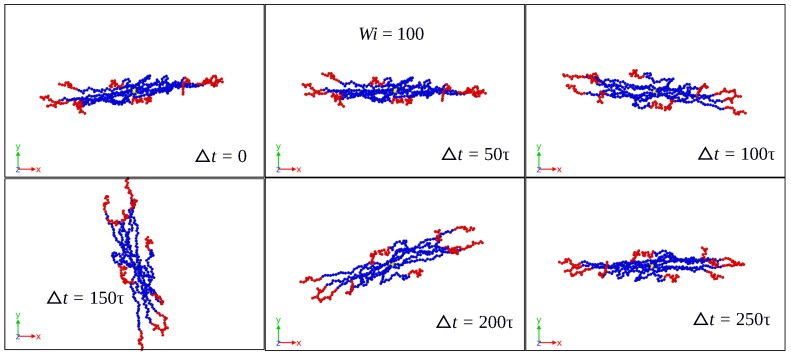
Time evolution of the (fixed) reference configuration in Eckart’s frame as seen in the laboratory frame for Case 1 and Wi=100.

**Figure 4:**
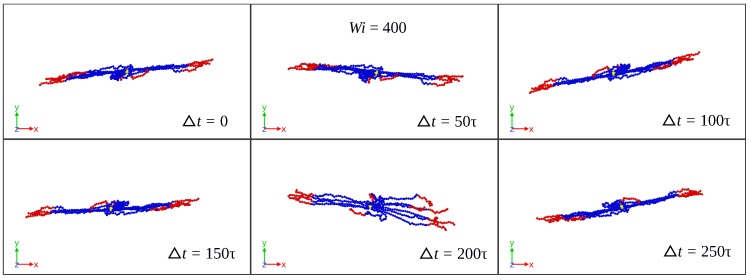
Time evolution of the (fixed) reference configuration in Eckart’s frame as seen in the laboratory frame for Case 1 and Wi=400.

Using the initial configuration of the SBC in the production run as the (fixed) reference configuration for Eckart’s frame, Figure 2, Figure 3 and Figure 4 show its time evolution as it is seen in the laboratory frame for Case 1 and different shear rates. For Wi=10, the reference configuration is seen in the lab frame as a rigid body rotating mainly around the vorticity axes. As the shear rate increases, the rotation takes place faster and around all three axes in lab frame, as illustrated by the cases Wi=100 and Wi=400. For the latter, Figure 3 and Figure 4 show a significant change of the Eckart frame orientation with respect to the lab frame. The polymer is expected to have a relatively high rotation frequency around the vorticity axis in the lab frame, which is found in the Eckart frame, as well (see Appendix A).

The angular velocity Ω of the Eckart coordinate system can be determined by starting from the time derivative of the Eckart condition [14,20,21]:

(14) $$M\sum_{k}c_{k}\times \rho_{k}=0.$$

Taking into account that the unit vectors of the Eckart frame evolve in time like rotations of a rigid body, ${\dot{f}}_{\mu }=\Omega \times f_{\mu }$, (μ=1,2,3), the Eckart angular velocity Ω is expressed as:

(15) $$\Omega =J^{\prime -1}L^{\prime },$$

where the ‘inertia tensor’ $J^{\prime }$ and the ‘angular momentum vector’ $L^{\prime }$ are given by the relations:

(16) $$J^{\prime }=M\sum_{k}\left[\left(\Delta r_{k}\cdot c_{k}\right)I-\Delta r_{k}⊗c_{k}\right]$$

and:

(17) $$L^{\prime }=M\sum_{k}c_{k}\times \Delta {\dot{r}}_{k}.$$

The above equations provide an expression for the (instantaneous) angular velocity Ω of rotation of the Eckart frame. Note that in the case of a truly rigid body, $\Delta r_{k}=c_{k}$ at all times, and thus, $J^{\prime }$ and $L^{\prime }$ become a true inertia tensor and angular momentum vector, respectively.

In this frame, the kinetic energy of the polymer can be written as (see Appendix B):

(18) $$E_{\mathrm{kin}}=\frac{1}{2}M_{s}\;{\dot{r}}_{\mathrm{cm}}\cdot {\dot{r}}_{\mathrm{cm}}+\frac{1}{2}\Omega \cdot \hat{J}\cdot \Omega +\frac{1}{2}M\sum_{k}{\tilde{v}}_{k}\cdot {\tilde{v}}_{k}+\frac{1}{2}M\sum_{k}u_{k}\cdot u_{k}+M\sum_{k}\left(\Omega \times c_{k}\right)\cdot u_{k},$$

where $\hat{J}$ is the inertia tensor using the Eckart variables (see Equation (20) below) and $u_{k}$ represents the angular contribution of the vibrational motion, i.e., the part of *k*-th monomer vibrational motion coupled to the rotations if the angular velocity is calculated by the (lab frame) standard approach. The last four terms of Equation (18) represent the kinetic energy contributions from, respectively, pure rotation, vibrations without angular momentum, vibrations with angular momentum and the Coriolis coupling (see Table 2). As can be seen, application of the Eckart frame formalism allows one to distinguish between vibrations without and with angular momentum contribution, the latter being displacements with respect to the pure rotation of the reference configuration [14].

#### 2.2.3. Hybrid Frame

As mentioned before, the introduction of the Eckart frame allows one to obtain an optimal separation of rotation and vibration. This feature has been employed in the formulation of symplectic integrators for MD simulations, which are applicable to molecules having one equilibrium configuration and which allows the evaluation of internal high-frequency vibrations [24,25,26,27]. Despite its success in describing the vibrational dynamics of small molecules, it is interesting to note that the definition of the inertia tensor for Eckart’s frame derived from the Eckart condition and given by Equation (16) does not meet in general the symmetry condition, i.e.,

(19) $$\Delta r_{k,\mu }c_{k,\nu }\neq \Delta r_{k,\nu }c_{k,\mu }.$$

To fulfil this last condition, we further explored a hybrid frame, in which we combine a proper, rigid-body inertia tensor $\hat{J}$ [22,23] with the deformable-body angular momentum L resolved on its Eckart-frame components, to define a new angular velocity W. In particular, we define:

(20) $${\hat{J}}_{\mu \nu }=M\sum_{k}\left[c_{k}^{2}\delta_{\mu \nu }-c_{k,\mu }c_{k,\nu }\right],$$

and the angular momentum (performing a transformation between the laboratory and Eckart’s frames [23]),

(21) $$\hat{L}=\sum_{\mu =1}^{3}\left(L\cdot f_{\mu }\right)f_{\mu }.$$

The angular velocity of the hybrid system is then given by the expression:

(22) $$W={\hat{J}}^{-1}\hat{L}.$$

In analogy with the expressions in the laboratory and Eckart frames, we also consider here a rotational kinetic energy:

(23) $$E_{\mathrm{rot}}=\frac{1}{2}W\cdot \hat{J}\cdot W.$$

#### 2.2.4. Geometrical Approach

A last, complementary approach to estimate the rotational frequency of soft colloids under shear is the so-called geometrical approximation (GA). This is based on two assumptions about the behavior of the polymers in linear shear flow [28,29]. First, it is assumed that the velocity of the monomers is entirely defined by the local, undisturbed velocity profile of the flow according to:

(24) $$\Delta {\dot{r}}_{k}≃\dot{\gamma }\Delta r_{k,y}{\hat{e}}_{1}.$$

Under this assumption, the instantaneous angular momentum of the polymer is given by the expression:

(25) $$L=M\sum_{k}\Delta r_{k}\times \Delta {\dot{r}}_{k}≃M_{s}\dot{\gamma }\left(G_{23}{\hat{e}}_{2}-G_{22}{\hat{e}}_{3}\right),$$

where $G_{\mu \nu }=N_{\mathrm{mon}}^{-1}\sum_{k}\Delta r_{k,\mu }\Delta r_{k,\nu }$ denotes the μν-component of the gyration tensor, which measures the overall conformation of the SBC. Furthermore, a long-time average is then performed in Equation (25), whereupon the non-diagonal element of the gyration tensor disappears, and thus, the average angular momentum has a single component, along the vorticity axis. Finally, it is assumed that the rotation of the SBC takes place mainly around the vorticity axis ${\hat{e}}_{3}$, i.e., $\omega_{1}=\omega_{2}\approx 0$. Within these approximations, $\omega_{3}=\omega_{G}$ has a constant value, and using Equation (7) it results in:

(26) $$\omega_{G}≃-M_{s}\dot{\gamma }\frac{\langle G_{22}\rangle }{\langle J_{33}\rangle }=-\dot{\gamma }\;\frac{\langle G_{22}\rangle }{\langle G_{11}\rangle +\langle G_{22}\rangle }.$$

Though clear by the construction of the GA, it is worth emphasizing once again that the so-obtained estimate for the angular frequency is a result of averaging the polymer motion over very long time intervals while at the same time making the a priori assumption that the instantaneous velocities of the monomers only have a component along the shear direction, dictated by the undistorted solvent velocity profile; see Equation (24). The final result, Equation (26), corresponds to the tumbling (rotation) frequency of a rigid body, the shape of which is similar to the average shape of the SBC and which also has an angular momentum equal to the value given by the mean flow [13,14]. At the same time, however, due to Equation (24), the estimate $\omega_{G}$ is also valid for a tank-treading (TT)-type of motion, in which the SBC does not rotate as a whole, but rather, the individual arms rotate by tank-treading around the geometrical star center, which remains at rest. This is a different, prototypical type of motion, for which the overall shape of the star remains fixed in time, i.e., no tumbling of the soft colloid as a whole takes place.

## 3. Results and Discussion

### 3.1. Global Conformation and Dynamics

As flexible polymers generically behave in shear flow, the SBC are stretched along the shear direction, compressed along the orthogonal (gradient and vorticity) directions and exhibit a preferred (average) orientation with respect to the flow. These global features are quantified by the average values of the gyration tensor G and the orientational angle $\chi_{G}$, both of which can be measured experimentally. The latter measures the flow-induced alignment of the polymer and is defined as the angle formed between the eigenvector ${\hat{g}}_{1}$ associated with the largest eigenvalue of G and the flow direction ${\hat{e}}_{1}$, and it can be evaluated as:

(27) $$\tan\left(2\chi_{G}\right)=\frac{2\langle G_{12}\rangle }{\langle G_{11}\rangle -\langle G_{22}\rangle }\equiv \frac{m_{G}}{Wi},$$

defining in this way the orientational resistance $m_{G}$ of the stars in shear flow.

**Figure 5:**
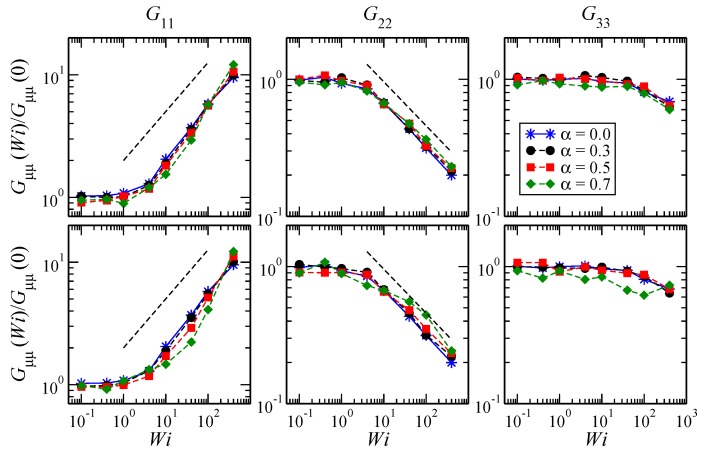
Diagonal components of the (average) gyration tensor of an SBC with f=18, $N_{\mathrm{pol}}=40$, λ=1.0 (top row) and λ=1.1 (bottom row). For athermal stars, the scalings $G_{11}∼Wi^{0.4}$ and $G_{22}∼Wi^{-0.32}$ are found at high Wi.

**Figure 6:**
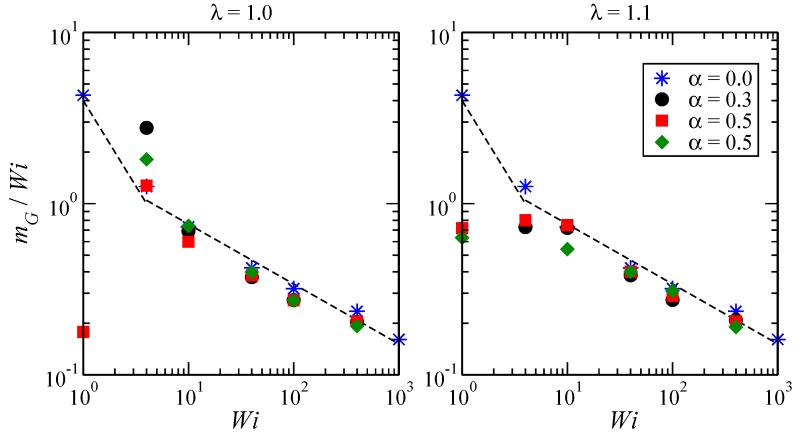
Reduced orientational resistance $m_{G}/Wi$ for stars with f=18, $N_{\mathrm{pol}}=40$ and values of λ and α as indicated on the panels. For athermal stars, we find $m_{G}/Wi∼Wi^{-0.83}$ at small Wi, and $m_{G}/Wi∼Wi^{-0.30}$ at large Wi.

At low values of Wi, the SBCs are hardly distorted, whereas for Wi≳10, they become increasingly anisotropic, expanding in the flow direction and shrinking most strongly in the shear direction and in minor proportion along the vorticity axis, as demonstrated by the diagonal components of the gyration tensor in Figure 5. Similarly, Figure 6 displays the average alignment angle as a function of the shear rate. At low shear rates (Wi<1), the scaling $\tan\left(2\chi_{G}\right)∼Wi^{-0.83}$ is found, while for Wi>10, it behaves as $\tan\left(2\chi_{G}\right)∼Wi^{-0.3}$, which is in agreement with previously-reported values [6].

**Figure 7:**
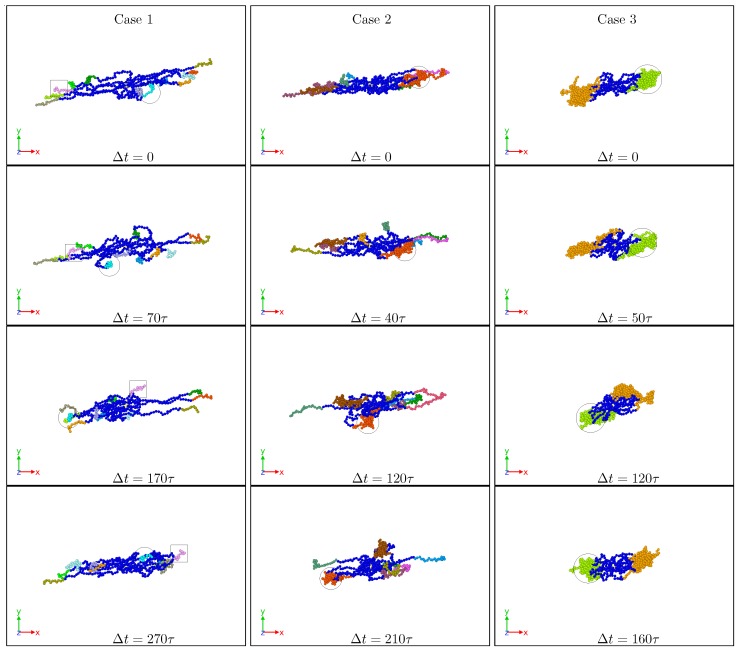
Representative simulation snapshots displaying the time evolution of the star block-copolymer (SBC) in shear flow for {f,α,λ}={12,0.3,1.0} (Case 1), {15,0.5,1.1} (Case 2) and {18,0.7,1.1} (Case 3). In Case 1, individual arms of the star perform tank-treading motion, while in Case 3, the star tumbles as a whole. Case 2 presents a tank-treading-like motion, but it is performed by both individual and clustered arms. Circles and squares are guides to follow the motion of arms. In all cases, $N_{\mathrm{pol}}=40$ and Wi=100. In the panels, Δt represents the elapsed time from the first configuration in τ units.

The overall (equilibrium) shape of an SBC depends on the number of patches formed and the compactness of the latter, which in turn depends on *f*, $N_{\mathrm{pol}}$, α and λ. Depending on the values of these parameters, three general cases can be recognized. At low α and λ (α<0.3 and λ<1.0), the star block copolymers behave very similarly to athermal stars (α=0) with no formation of patches or very weak, breakable ones (Case 1). On the other opposite limit, at high α and λ (α≳0.6 and λ≳1.1), the macromolecule acquires cylindrical symmetry around its principal axis, since it self-assembles into dumbbell-like structures with one or two massive patches (Case 3). At intermediate values of α and λ, the SBCs form a number of patches that can break-up and/or merge as a consequence of shear (Case 2) [10,11,12]. These three tendencies can be also observed from the dynamical point of view, as displayed in Figure 7, where characteristic snapshots are shown, helping to visualize the time evolution of the SBCs under shear. As can be seen there, for low amphiphilicity and good solvent, the SBC behaves in a similar way as athermal stars, and then, the arms perform tank-treading-like (TT) motions. As the contribution of the attractive interaction increases, patches begin to form and TT rotation is also found, but this time, the motion is simultaneously performed by all arms forming the cluster. Finally for high α and λ, the SBC motion closely resembles that of a rigid dumbbell. We will explore, in what follows, the ways in which these statements based on impressions from simulation snapshots acquire quantitative character through the comparison of characteristic quantities among different reference frames and approximations.

### 3.2. Reference Configuration Update

In the original Eckart formalism, the rigid reference configuration of (small) molecules is assumed to be the equilibrium one (all forces on all monomers vanishing), and its dynamics is governed by the time evolution of the positions of the atoms forming the molecule, which are defined by vectors $c_{k}$; see Equation (13). Since thermally-fluctuating (star) polymers do not have such a rigid equilibrium configuration, but rather a multitude of typical configurations related to the given conditions (temperature and shear rate), it is plausible to think that, as the simulation advances, the reference configuration needed to build up the Eckart frame must be updated at regularly-spaced numbers of MD steps. The period of updating the characteristic, reference configuration is denoted as $t_{\mathrm{Eckart}}$, and it can vary at will, from a very frequent update of the reference configuration that tries to follow the details of the particle motion to a rare one, for which the average, time-coarsened rotational dynamics of the molecule is captured.

**Figure 8:**
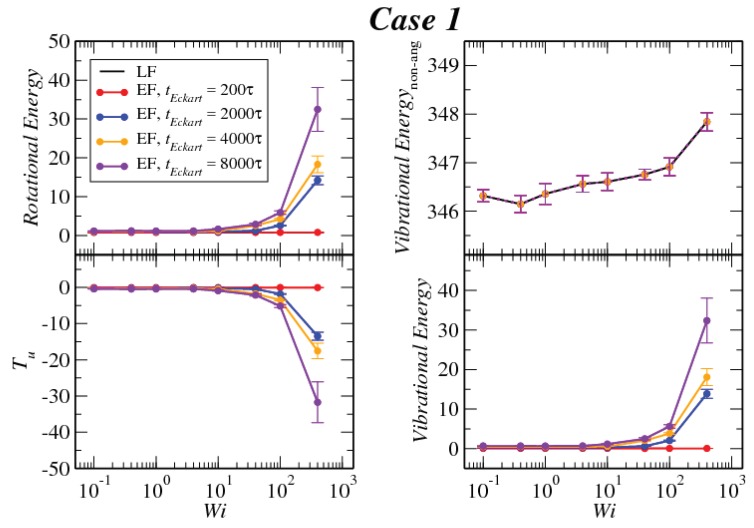
Comparison between the values of the kinetic energy for Case 1 evaluated in both the lab and Eckart frames at different Eckart times (Table 2).

**Figure 9:**
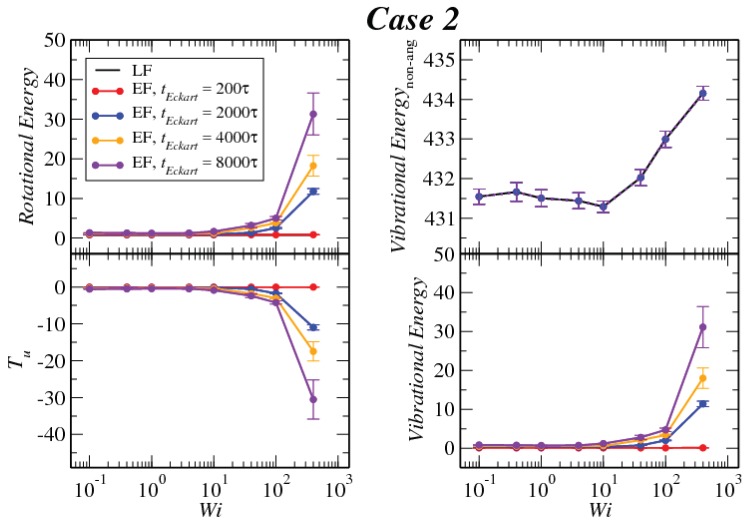
Comparison between the values of the kinetic energy for Case 2 evaluated in both the lab and Eckart frames at different Eckart’s times (Table 2).

**Figure 10:**
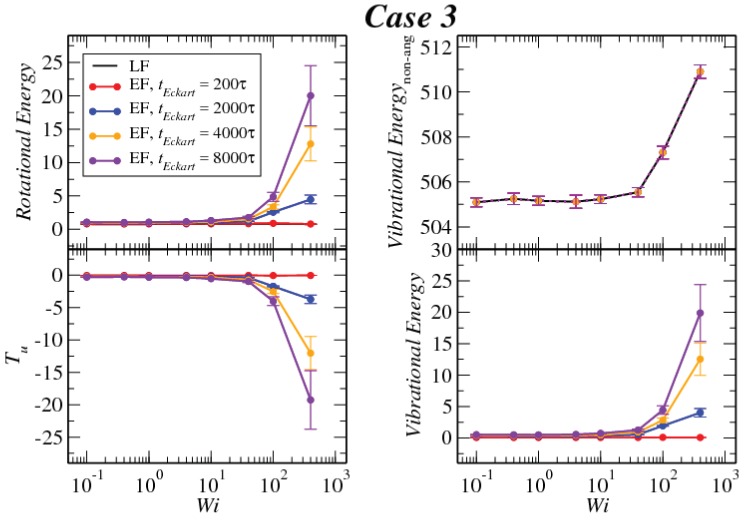
Comparison between the values of the kinetic energy for Case 3 evaluated in both the lab and Eckart frames at different Eckart’s times (Table 2).

In Figure 8, Figure 9 and Figure 10, we compare the behavior of the different contributions to the kinetic energy (see Table 2) as a function of the Weissenberg number for different values of $t_{\mathrm{Eckart}}$. For $t_{\mathrm{Eckart}}=200\;\tau$, the rotational energy grows very slowly with Wi (it is essentially constant), and it coincides with the value that it obtains in the laboratory. In this case, where the reference configuration is updated very frequently, the rotational frequencies ω and Ω in the LF and the EF are very similar, i.e., ω≃Ω and also $\hat{J}≃J$, resulting in the approximate equality of rotational energies:

(28) $$\frac{1}{2}\omega \cdot J\cdot \omega ≃\frac{1}{2}\Omega \cdot \hat{J}\cdot \Omega$$

Related to this approximate equality is the vanishingly small value of the kinetic energy contribution $T_{u}$, which emerges as the sum of the angular-momentum-carrying contributions and the Coriolis coupling, viz.:

(29) $$T_{u}=\frac{M}{2}\sum_{k}u_{k}\cdot u_{k}+M\sum_{k}\left(\Omega \times c_{k}\right)\cdot u_{k}.$$

The reason for the smallness of this term lies in that the quantity $u_{k}$ itself is small. Indeed, since $u_{k}=\omega \times \Delta r_{k}-\Omega \times c_{k}$, the proximities of angular velocities and configurations ($\Delta r_{k}≅c_{k}$) imply the smallness of $u_{k}$ and of both terms in the right-hand side of Equation (29) above. Another useful way to look into the quantity $T_{u}$ is to express it as (see Appendix C):

(30) $$T_{u}=\frac{1}{2}\omega \cdot J\cdot \omega -\frac{1}{2}\Omega \cdot \hat{J}\cdot \Omega .$$

Evidently, $T_{u}$ is the difference in the rotational energies between the LF and EF, and its small value affirms the similarity of the two for frequent updates of the reference configuration in the Eckart frame.

**Figure 11:**
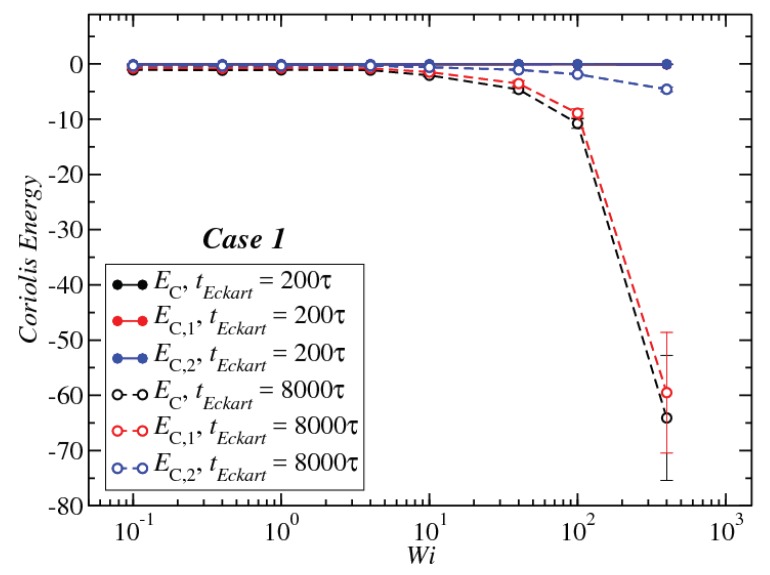
The Coriolis coupling for two different values of $t_{\mathrm{Eckart}}$ for Case 1 as a function of Wi. For the meaning of the quantities $E_{C}$, $E_{C,1}$ and $E_{C,2}$, see Equation (31).

Upon increasing the time intervals between updates of the reference configuration, deviations between the LF and the EF appear in the strongly nonlinear regime, Wi>10. The EF rotational energy grows much higher than its LF counterpart, signaling significant deviations between the (temporally coarse) EF angular velocity Ω and its LF-counterpart ω. This phenomenon is consistently accompanied by an increase in the magnitude of $T_{u}$, as well as an increase in the magnitudes of the velocities $u_{k}$, leading to a growth of the angular-momentum carrying vibrational parts of the energy. The second term on the right-hand side of Equation (29) is the Coriolis term $E_{C}$, which can be rewritten in the form:

(31) $$\begin{array}{ccc} E_{C} & = & E_{C,1}+E_{C,2}\\ M\sum_{k}\left(\Omega \times c_{k}\right)\cdot u_{k} & = & -M\sum_{k}\left(\Omega \times \rho_{k}\right)\cdot u_{k}+M\sum_{k}\left(\Omega \times \Delta r_{k}\right)\cdot u_{k}, \end{array}$$

defining the partial terms $E_{C,1}$ and $E_{C,2}$ with the help of the vector $\rho_{k}=\Delta r_{k}-c_{k}$, Equation (A1). The behavior of each term of the Equation (31) is shown in Figure 11 only for Case 1 as representative for all other cases, as well. For $t_{\mathrm{Eckart}}=200\;\tau$, the Coriolis coupling is close to zero, but for $t_{\mathrm{Eckart}}=400\;\tau$, the Coriolis coupling is negative, and the contribution related to $\rho_{k}$, the second term in the right of Equation (31), is dominant in the Coriolis coupling behavior.

Finally, the vibrational kinetic energy associated with the velocities carrying no angular momentum, $E_{\mathrm{vib}}=\left(M/2\right)\sum_{k}{\tilde{v}}_{k}\cdot {\tilde{v}}_{k}$, is very large, and its value is essentially independent of $t_{\mathrm{Eckart}}$: the stars have a large number of breathing and fast oscillatory modes. Even for the case of short Eckart times, for which the quantities $\rho_{k}$ and $u_{k}$ are small, the quantities ${\dot{\rho }}_{k}={\tilde{v}}_{k}+u_{k}≃{\tilde{v}}_{k}$ are significant and denote fast oscillations of the corresponding displacement variables.

### 3.3. Angular Momentum and Angular Frequency

**Figure 12:**
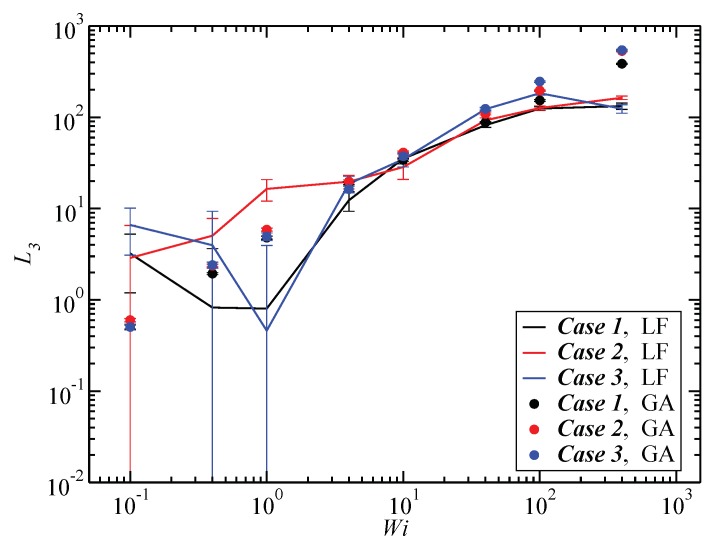
Comparison between the exact value of the component $L_{3}$ of the angular momentum, Equation (4), with the one obtained from the geometrical approximation, Equation (25).

We now proceed to our results regarding the angular momenta and frequencies of the SBC motions under shear flow. In Figure 12, we compare the component of the total angular momentum around the vorticity direction $L_{3}$ in the laboratory frame from Equation (4) to the value evaluated through the geometric approximation, Equation (25). The velocity of the monomers for intermediate values of Wi is well approximated by Equation (24), i.e., it is mainly determined by the velocity of the fluid, at least in the average sense.

**Figure 13:**
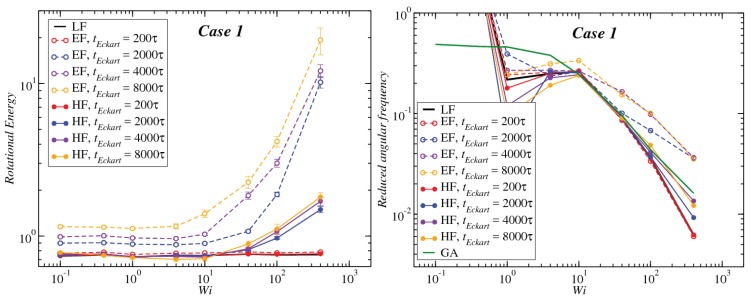
Left panel: Rotational energy ($\mathrm{LF}\to \frac{1}{2}\omega \cdot J\cdot \omega$, $\mathrm{EF}\to \frac{1}{2}\Omega \cdot \hat{J}\cdot \Omega$, $\mathrm{HF}\to \frac{1}{2}W\cdot \hat{J}\cdot W$) for the different frames as a function of the Weissenberg number Wi. Right panel: reduced angular frequency for Case 1 ($\mathrm{LF}\to \omega /\dot{\gamma }$, $\mathrm{EF}\to \Omega /\dot{\gamma }$, $\mathrm{HF}\to W/\dot{\gamma }$, $\mathrm{GA}\to \omega_{G}/\dot{\gamma }$) as a function of Wi for different Eckart times.

**Figure 14:**
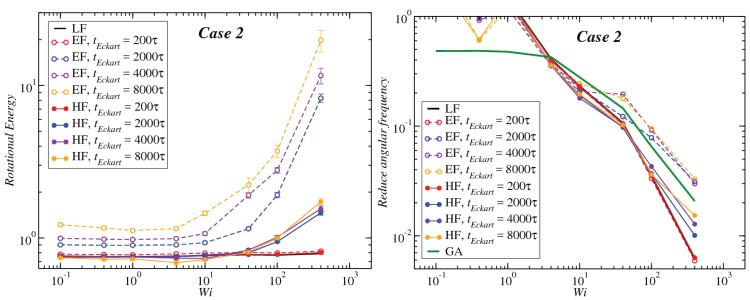
Same as Figure 13, but for Case 2.

**Figure 15:**
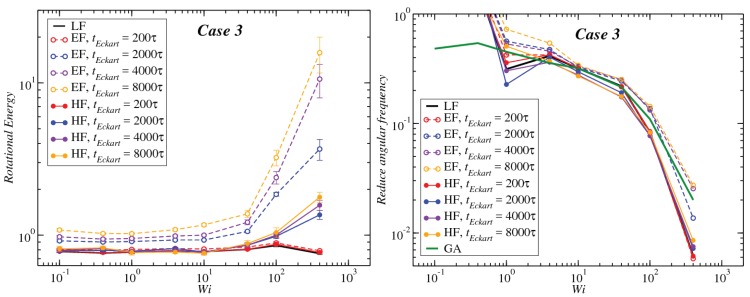
Same as Figure 13, but for Case 3.

Results for the angular frequency as a function of Wi and the dependence of this function on the frame of reference, as well as on the configuration update time $t_{\mathrm{Eckart}}$ are shown in Figure 13, Figure 14 and Figure 15, right panels. According to our analysis, since the block copolymer stars under consideration are very soft systems, the frequency of rotation in the Eckart frame should be closer to the geometrical approach, and therefore, one would expect that the decay law for high Wi should be the same in both approximations for sufficiently long updating intervals $t_{\mathrm{Eckart}}$. Our findings confirm that, indeed, the Eckart rotation frequencies lie closer to those from the geometric approximation, and they have the ones obtained by the laboratory frame analysis as a lower bound. As $t_{\mathrm{Eckart}}$ grows, the Eckart rotation frequencies move from the LF towards and beyond the GA curves, confirming the fact that at coarse time scales, the stars, at least for Cases 1 and 2, can be thought of as soft colloids with a tank-treading type of motion of the polymers in their interior.

**Figure 16:**
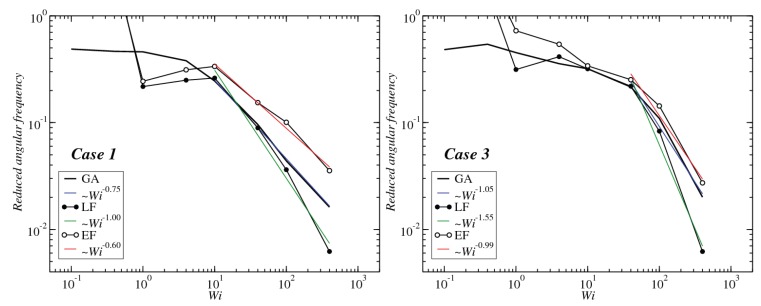
The reduced angular frequencies for Case 1 (**left**) and Case 3 (**right**) evaluated at the LF, the GA and the EF with the longest Eckart time employed, $t_{\mathrm{Eckart}}=8000\;\tau$.

Case 3 seems exceptional, in the sense that the angular frequency evaluated in the EF appears to be almost independent of the parameter $t_{\mathrm{Eckart}}$ and always very close to the GA result. This is an indication of the fact that, contrary to the other two cases, these star block copolymers do not behave as tank-treading soft colloids. On the contrary, and consistent with their rather compact, elongated, dumbbell-shape, they rotate similarly to rigid prolate ellipsoids under constant shear flow. In particular, the GA-assumption of isolated monomers, each of which is carried through the solvent with the local velocity of the streaming solvent, are responsible for giving these molecules the character of rigid-like, stiff objects, as opposed to the very soft and flexible polymers of Case 1, for which associations among the end-monomers are rare and easily breakable. To emphasize the difference between Case 1 and Case 3, in Figure 16, we plot the angular frequencies for the two limiting frames, LF and GA, together with the EF result at the longest Eckart time, $t_{\mathrm{Eckart}}=8000\;\tau$. As can be seen, whereas for Case 1, the EF frequencies exceed both the LF and the GA ones, for Case 3, EF and GA are very close to one another. Differences in the power-law behavior for large values of Wi between the two cases can also be seen.

## 4. Conclusions

In this work, we analyzed the rotational dynamics of an isolated star-shaped block copolymer under shear flow for three representative sets of parameters, i.e., a very flexible system (Case 1), an intermediate flexible-rigid system (Case 2) and, finally, a rather rigid system (Case 3). Motivated by very recent studies on polymer dynamics [13,14], we explored the quantitative predictions emerging from the employ of the Eckart frame formalism and compare them to the resulting ones from two different approaches (lab frame and geometrical approach). Additionally, we performed an analysis of each term in the kinetic energy and the contributions of the various kinetic terms to it.

In addition to the standard Eckart formalism [22], extended to polymers under flow in [14,20,21], we suggested a “hybrid” definition of the rotation frequency. As a consequence, we obtained different analytical approximations for the total kinetic energy and for the numerical value for the rotational frequency of the SBC, which we express using strictly the Eckart’s variables. It is important to note that both treatments reproduce correctly the results for the laboratory frame for small updating time $t_{\mathrm{Eckart}}$ ($t_{\mathrm{Eckart}}∼200\tau$); however, for $t_{\mathrm{Eckart}}>200\tau$, we found differences between both treatments, particularly for the rotational energy term. For Wi<10, we found that the rotational energy is independent of $t_{\mathrm{Eckart}}$ in the hybrid formulation, which is not the case for the rotational energy associated with the Eckart rotational frequency. Additionally, both the rotational energy and frequency found in [14] are larger than the outcomes from the hybrid treatment.

The main result concerns the behavior of the associated rotational frequency Ω at high shear rates (Wi>100) for the three different systems. We found that for all cases, Ω is bounded from below by the rotational frequencies obtained in the lab frame (ω). For the third case, i.e., self-assembled, dumbbell-like SBC, $\Omega \approx \omega_{G}$ for sufficiently large values for the updating time $t_{\mathrm{Eckart}}$, demonstrating that the rotation frequency mainly corresponds to tumbling motion of the SBC induced by the shear flow. On the other hand, for Case 1, which is closely related to athermal star polymers, the results obtained from the geometrical approximation are consistent with the Eckart frame only for long enough $t_{\mathrm{Eckart}}$; therefore, the geometrical approximation only captures the average, time-coarsened tank-treading rotational frequency of the polymer. These results agree with those obtained for athermal stars with smaller polymerization degree ($N_{\mathrm{pol}}=6$), for which it was found that the vibrational angular momentum has a larger contribution for softer polymers [14].

The dynamics of Case 2 is richer; although this system features four patches on average [12], the shear causes those patches to break and to cluster over and over again. Therefore, here, the rotational frequency results from the average of the tank-treading motion of free and clustered arms. It remains to establish a more detailed description regarding the statistic of the typical times between break-up and rejoin events, which shed light on their influence on the rheology of semi-dilute suspensions, in particular on the expected shear thinning behavior and how it can be tuned by the amphiphilicity and the solvent quality [1].

## Figures and Tables

**Table 1:** List of shorthands and abbreviations for systems and methods used in this work.

Abbreviation	Meaning
Case 1	{f,α,λ}={12,0.3,1.0}
Case 2	{f,α,λ}={15,0.5,1.1}
Case 3	{f,α,λ}={18,0.7,1.1}
LF	Laboratory frame
EF	Eckart frame
HF	Hybrid frame
GA	Geometric approximation

**Table 2:** The various contributions to the total kinetic energy in the laboratory, the Eckart frames and hybrid frame.

	Rotational	Vibrational without Angular Momentum	$T_{u}$ = Vibrational with Angular Momentum + Coriolis Coupling
Laboratory Frame	$\frac{1}{2}\omega \cdot J\cdot \omega$	$\frac{M}{2}\sum_{k}{\tilde{v}}_{k}\cdot {\tilde{v}}_{k}$	–
Eckart frame	$\frac{1}{2}\Omega \cdot \hat{J}\cdot \Omega$	$\frac{M}{2}\sum_{k}{\tilde{v}}_{k}\cdot {\tilde{v}}_{k}$	$\frac{M}{2}\sum_{k}u_{k}\cdot u_{k}+M\sum_{k}\left(\Omega \times c_{k}\right)\cdot u_{k}$
Hybrid frame	$\frac{1}{2}W\cdot \hat{J}\cdot W$	$\frac{M}{2}\sum_{k}{\tilde{v}}_{k}\cdot {\tilde{v}}_{k}$	–

**Figure A1:**
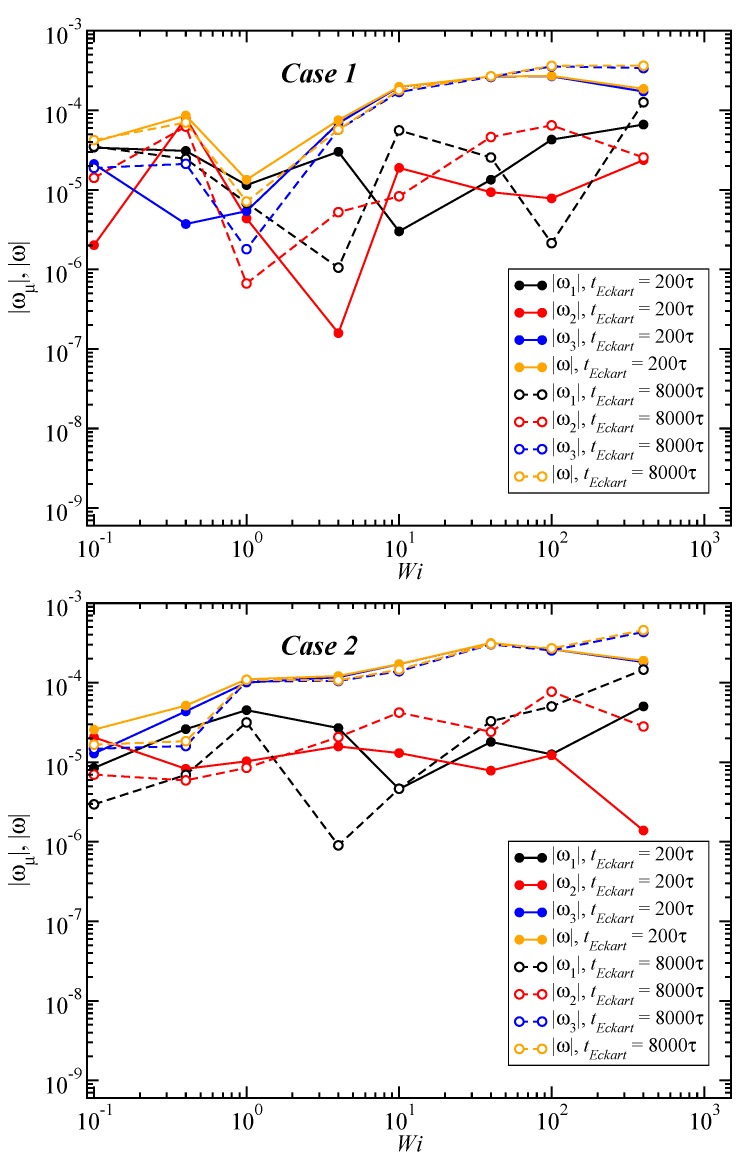

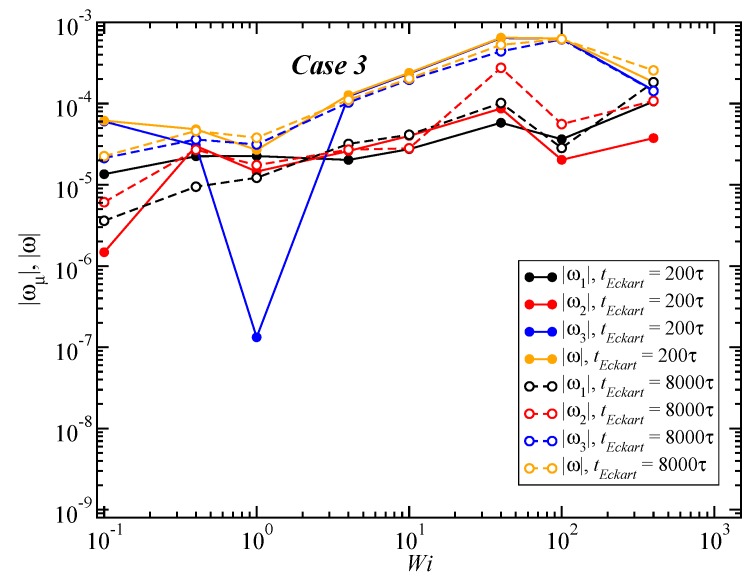
Values of the average magnitudes of the components of the angular velocity, $|\omega_{\mu }|$, and the magnitude of the whole vector, |ω|, for Case 1 as a function of Wi for two different values of $t_{\mathrm{Eckart}}$ as indicated in the legend, for Cases 1, 2 and 3.

## Appendix A. Rotation Frequencies

In Figure A1, we show results for all components of the angular frequency. In general, we find that the angular velocity in the vorticity axis is dominant in the angular frequency vector, especially as Wi grows. The vorticity component $\omega_{3}$ approaches a constant value at high values of Wi or even shows a decrease there, in Case 3.

## Appendix B. Kinetic Energy in the Eckart Frame

The definition of the displacement vector ρ compatible with the Eckart frame [14,22,23] reads as:

(A1) $$\rho_{k}=\Delta r_{k}-c_{k},$$

Taking the time derivative, we find the equation of motion:

(A2) $${\dot{r}}_{k}={\dot{r}}_{\mathrm{cm}}+\left(\Omega \times c_{k}\right)+{\tilde{v}}_{k}+u_{k}.$$

We note that for $t_{\mathrm{Eckart}}=200\;\tau$, ω≈Ω, $\Delta r_{k}\approx c_{k}$ and $u_{k}\approx 0$ (see Section 3.2). Concomitantly with Equation (A2), the resulting kinetic energy is:

(A3) $$\begin{array}{ccc} \frac{M}{2}\sum_{k}{\dot{r}}_{k}\cdot {\dot{r}}_{k} & = & \frac{M_{s}}{2}{\dot{r}}_{cm}\cdot {\dot{r}}_{cm}+\frac{M}{2}\sum_{k}\left(\Omega \times c_{k}\right)\cdot \left(\Omega \times c_{k}\right)+\frac{M}{2}\sum_{k}{\tilde{v}}_{k}\cdot {\tilde{v}}_{k}+\frac{M}{2}\sum_{k}u_{k}\cdot u_{k}+\\ & & {\dot{r}}_{cm}\cdot \left(\Omega \times M\sum_{k}c_{k}\right)+{\dot{r}}_{cm}\cdot M\sum_{k}{\tilde{v}}_{k}+{\dot{r}}_{cm}\cdot M\sum_{k}u_{k}+\\ & & M\sum_{k}\left(\Omega \times c_{k}\right)\cdot {\tilde{v}}_{k}+M\sum_{k}\left(\Omega \times c_{k}\right)\cdot u_{k}+M\sum_{k}{\tilde{v}}_{k}\cdot u_{k}. \end{array}$$

Since the following equalities hold [22,23],

(A4) $$M\sum_{k}c_{k}=0,$$

(A5) $$M\sum_{k}\Delta {\dot{r}}_{k}=M\Omega \times \sum_{k}c_{k}+M\sum_{k}{\tilde{v}}_{k}+M\sum_{k}u_{k}=0\to M\sum_{k}u_{k}=0,$$

and the definition of $u_{k}$ implies:

(A6) $$M\sum_{k}{\tilde{v}}_{k}\cdot u_{k}=M\sum_{k}{\tilde{v}}_{k}\cdot \left(\omega \times \Delta r_{k}\right)-M\sum_{k}{\tilde{v}}_{k}\cdot \left(\Omega \times c_{k}\right)=-M\sum_{k}{\tilde{v}}_{k}\cdot \left(\Omega \times c_{k}\right),$$

then the kinetic energy can be expressed as in Equation (18).

## Appendix C. Explicit Calculation of *T_u_*

Starting with the definition of vector $u_{k}$, Equation (A2), the energy $T_{u}$ (Table 2) can be written as,

(A7) $$\begin{array}{ccc} T_{u} & = & \frac{M}{2}\sum_{k}u_{k}\cdot u_{k}+M\sum_{k}\left(\Omega \times c_{k}\right)\cdot u_{k}\\ & = & \frac{M}{2}\sum_{k}\left(\omega \times \Delta r_{k}-\Omega \times c_{k}\right)\cdot \left(\omega \times \Delta r_{k}-\Omega \times c_{k}\right)+M\sum_{k}\left(\Omega \times c_{k}\right)\cdot \left(\omega \times \Delta r_{k}-\Omega \times c_{k}\right). \end{array}$$

Grouping in a convenient way,

(A8) $$T_{u}=\frac{1}{2}\left(\omega \cdot J\cdot \omega +\Omega \cdot \hat{J}\cdot \Omega -2M\left(\omega \times \Delta r_{k}\right)\cdot \left(\Omega \times c_{k}\right)\right)+\left(M\left(\omega \times \Delta r_{k}\right)\cdot \left(\Omega \times c_{k}\right)-\Omega \cdot \hat{J}\cdot \Omega \right),$$

so that:

(A9) $$T_{u}=\frac{1}{2}\omega \cdot J\cdot \omega -\frac{1}{2}\Omega \cdot \hat{J}\cdot \Omega .$$
